# A novel triazolonaphthalimide induces apoptosis and inhibits tumor growth by targeting DNA and DNA-associated processes

**DOI:** 10.18632/oncotarget.16962

**Published:** 2017-04-08

**Authors:** Liyan Ji, Simin Yang, Shasha Li, Shan Liu, Shunan Tang, Zhongqiu Liu, Xiangbao Meng, Siwang Yu

**Affiliations:** ^1^ Department of Chemical Biology, Peking University School of Pharmaceutical Sciences, Beijing 100191, China; ^2^ International Institute for Translational Chinese Medicine, Guangzhou Traditional Chinese Medicine University, Guangzhou 510006, China

**Keywords:** triazolonaphthalimide, DNA binding, topoisomerase 2, DNA replication, cell cycle arrest

## Abstract

DNA and DNA-associated processes have been classes of the most important targets of chemotherapeutic drugs. As classic DNA intercalators and topoisomerase inhibitors, naphthalimides have been extensively investigated as potential anti-cancer drugs. We recently synthesized a novel series of triazolonaphthalimides with excellent anti-cancer activities. In the present study, one of the most potent triazolonaphthalimides, LSS-11, was investigated. LSS-11 bound to DNA *in vitro* and *in cell* mainly by minor groove binding and significantly increased the stability of DNA, which could be fundamental for the biological activities of LSS-11. In addition to inhibiting DNA topoisomerase II-catalyzed decatenation of knotted circulated DNA, LSS-11 dramatically inhibited DNA replication mediated by polymerase chain reaction and isothermal helicase-dependent amplification, as well as the expression of luciferase driven by a minimal TA promoter *in cell*. Furthermore, LSS-11 exhibited strong cytotoxicity in selected human colon cancer cell lines by inducing cell cycle arrest and apoptosis, which was accompanied by DNA damage response. Finally, LSS-11 potently inhibited the growth of S180 murine sarcoma and SW480 human colorectal cancer xenografts *in vivo* without significant major toxicities. These results suggest that LSS-11 deserves further research and development as a novel anti-cancer agent, and provided new understandings of mechanisms by which LSS-11 inhibited multiple DNA-associated processes and tumor growth.

## INTRODUCTION

The integrity of the structure and functions of DNA is crucial for cell survival and proliferation. Cancer cells are more susceptible to perturbation in DNA structure and functions due to higher replication and transcription demands, relaxed DNA damage sensing and repair capability, and loose cell cycle checkpoint control [1]. Therefore, DNA has been one of the most intensively exploited targets of anti-cancer therapies from the very beginning. In fact, DNA has been successfully targeted by mechlorethamine to treat cancers even before the discovery of DNA double helix structure [2]. Since the approval of nitrogen mustard as cytotoxic anti-neoplasm agent, DNA-targeting agents such as antimetabolites, alkylating agents and platinum complexes have been successfully developed and clinically utilized for more than half a century [3]. In addition to agents that directly interact with DNA, small molecules targeting DNA-processing enzymes such as topoisomerases (Topo) and DNA repair enzymes have also been extensively investigated and proceeded into clinic [4]. Certain cancer stem cells possess a potent DNA damage response (DDR) system which contributes to resistance to conventional chemotherapy, and DDR was also observed in peritumoral regions and may promote tumor progression [5]. Therefore, targeting DDR in cancer cells or associated cells is an attractive strategy [6]. To the date, DNA-targeting agents still attract enormous research interests as anticancer therapeutics, and DNA-binding agents are the most explored and best characterized ones.

Naphthalimide (benz[de]isoquinoline-1,3-dione) derivatives are classical DNA intercalators that exhibit interesting fluorescent and a wide range of pharmacological properties, especially anti-cancer activities [7, 8]. It is generally believed that naphthalimides bind DNA by intercalation through their aromatic tricyclic planar heterocycles [9], but other binding patterns have also been suggested [8]. Substitutions on the rings further modulate their interactions with DNA, and alterations in the structure, conformation or other properties of DNA will have profound impact on multiple DNA-associated processes [8]. Many naphthalimides have been intensively investigated as anti-cancer agents, and several of them, such as amonafide, mitonafide, UNBS5162 several other napthalimides, have successfully entered clinical trials [9, 10]. However, to the date none of these compounds have succeeded to achieve market, mainly due to undesired toxicities and therapeutic efficacy. Inhibition of topoisomerases, especially topoisomerase II (Topo II) has been proposed as the major mechanism by which naphthalimides induce cell cycle arrest and apoptosis in cancer cells [11]. Other mechanisms have also been suggested to be involved in the anticancer effects of naphthalimides, including inhibition of histone deacetylase [12], DNA and RNA synthesis [13], receptor tyrosine kinases [14], NF-κB signaling [14], poly(ADP-ribose) polymerase-1(PARP-1) [15], thioredoxin reductase (TrxR) [16], restoration of the functions of p53 and p21 [17], induction of ROS and malfunction of lysosome and mitochondria [17–19]. However, the exact mechanisms by which naphthalimides impact cellular physiological processes remain largely in vague, and lack of detailed mechanistic information further dampened the attempts to improve the therapeutic efficacy and toxicological profiles of naphthalimides.

**Figure 1:**
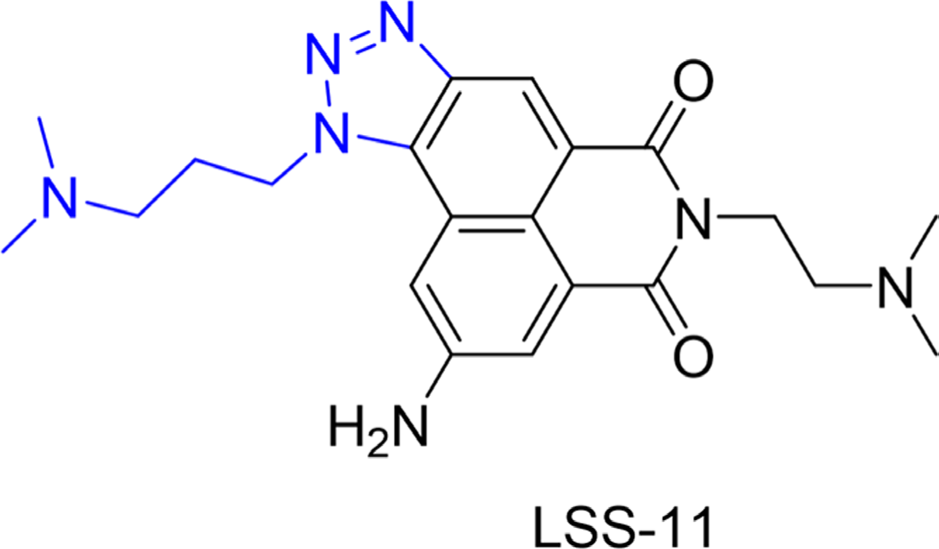
The chemical structures of LSS-11 The amonafide core structure is indicated by black line, while the triazole and tertiary amine side chain is in blue line.

A vast of efforts have been devoted to synthesize and evaluate novel naphthalimide derivatives with potential anticancer activities since the discovery of amonafide [20]. Different strategies have been employed to improve the efficacy and toxicological profiles [21], and one of the most attractive strategies to improve the DNA-binding affinity and anticancer activity of naphthalimides is to expand the aromatic ring system and to add basic side chains [22, 23]. For example, substitution by triazole moiety enhanced the cytotoxicity of naphthalimides [24]. Recently we successfully fused triazole ring to the naphthalene core by a novel method to obtain triazolonaphthalimides with significantly improved anti-cancer activities compared to triazol-substituted ones [25]. Then we further synthesized a series of triazolenaphthalimide derivatives and found one of these compounds, LSS-11 (2-amino-5-(2-(dimethylamino) ethyl)-10-(3-(dimethylamino)propyl) benzo [*de*] [1–3] triazole [4,5-g] isoquinoline-4,6 (5*H*,10*H*)- dione, Figure 1), exhibited outstanding cytotoxicity in a panel of cancer cell lines [26].

In the present study, the *in vitro* and *in vivo* anti-cancer activities of LSS-11 were tested, and the potential molecular mechanisms were investigated. Furthermore, the interaction of LSS-11 with DNA and its impact on several DNA-associated biological processes were examined. Finally, the *in vivo* efficacy of LSS-11 against S180 murine sarcoma and SW480 human colorectal carcinoma xenograft and its toxicological profile were further evaluated.

## RESULTS

### LSS-11 interacts with DNA and increases its stability

**Figure 2:**
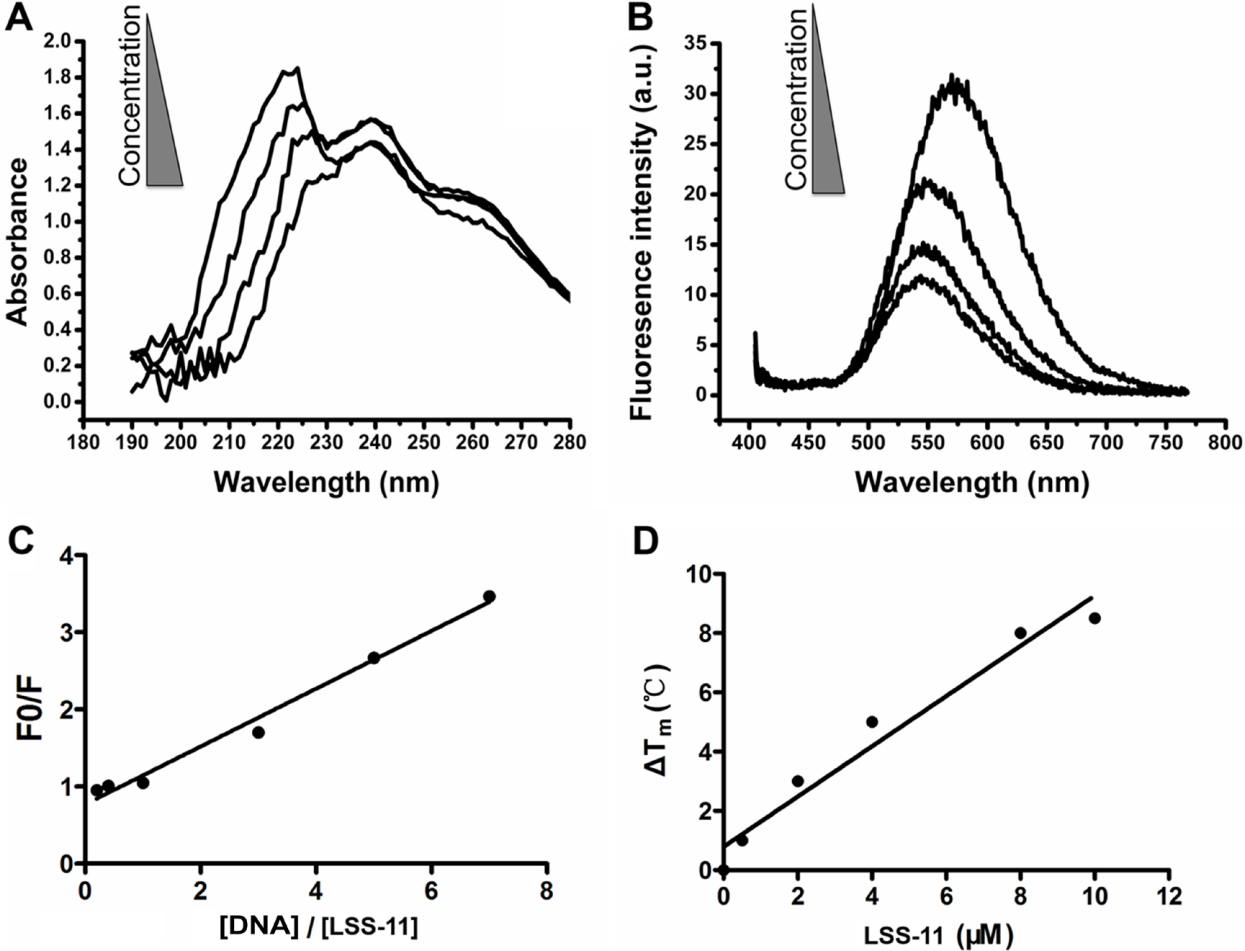
LSS-11 interacts with DNA and increases its stability (**A**) UV-Vis spectra and (**B**) fluorescent emission spectra of LSS-11 (50 μM) with increasing concentrations of CT DNA (0 to 200 μM). (**C**) Scatchard plot of the fluorescent intensity of LSS-11 at 570 nm with increasing concentrations of CT DNA [DNA], F stands for fluorescent intensity and F0 refers to fluorescent intensity without CT DNA. (**D**) Increased Tm of a 63 bp DNA fragment in the presence of LSS-11 at indicated concentrations.

The interaction of LSS-11 with DNA was firstly characterized by ultraviolet-visible and fluorescent spectrometry. As shown in Figure 2A, addition of CT DNA into LSS-11 solution concentration-dependently induced a red shift and reduction of its peak absorbance around 220 nm. CT DNA also concentration-dependently quenched the fluorescence emission of LSS-11 around 570 nm (Figure 2B). The apparent binding constant was calculated to be 2.5 × 10^5^ M^-1^ by using Wolfe's model [27] (Figure 2C). These results indicate that LSS-11 potently interacted with DNA. Furthermore, the fluorescence of LSS-11 could also be quenched by RNA at similar concentration to that of DNA (Supplementary Figure 1).

Usually binding of ligands to DNA stabilizes the base pair stacks, which will change the Tm of DNA. As expected, LSS-11 increased the TM of a 63 bp DNA fragment, which shows that LSS-11 binding significantly enhanced the stability of DNA double strand. In the presence of 10 μM LSS-11, the TM of the DNA fragment increased by 8°C (Figure 2D), indicating significantly enhanced stability. On the other hand, the presence of etoposide (Topo II inhibitor) or SN-38 (Topo I inhibitor) did not change the TM of DNA (data not shown). Please note that the excitation and emission wavelengths of SYBR green (497 nm and 520 nm, respectively) are different from that of LSS-11 (386 nm and 470 nm, respectively).

### LSS-11 binds DNA *in vitro* and in cell by minor groove binding

**Figure 3:**
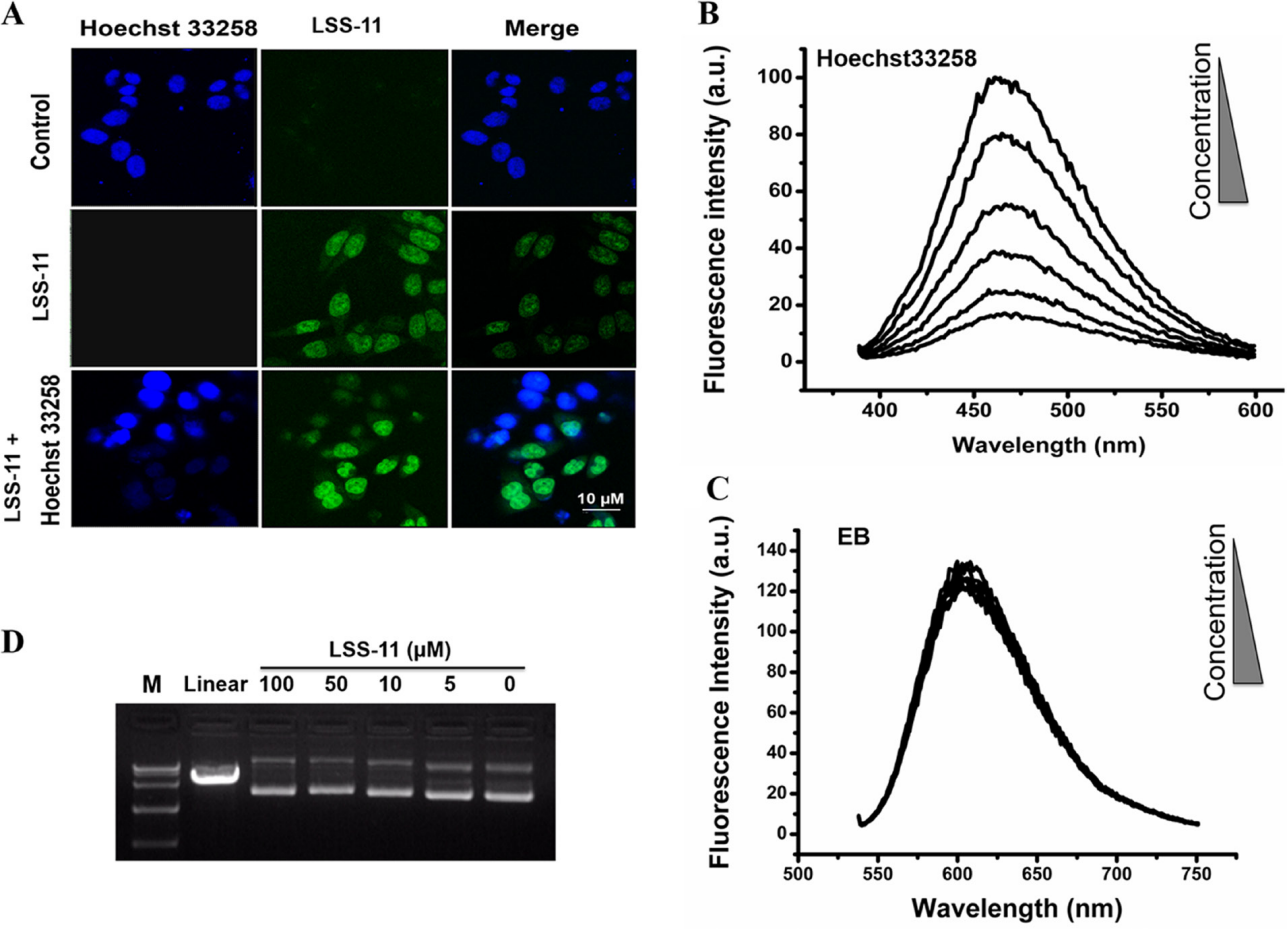
LSS-11 binds DNA *in vitro* and in cell by minor groove binding (**A**) Fluorescent microscopic photography showing cells stained by Hoechst33258 (blue), LSS-11 (green) or both. Please note the mutual exclusive distribution of Hoechst33258 and LSS-11 fluorescence in nucleus. (**B**) and (**C**) Competitive fluorescent spectra titration of LSS-11 to Hoechst33258 (B) or EB (C). (**D**) Agarose electrophoresis of pUC-19 plasmid incubated with indicated concentrations of LSS-11 and stained with EB, pUC-19 digested by endonuclease was employed as a positive control.

Naphthalimides have been reported to bind DNA mainly by intercalation, but minor groove binding has also been reported [28]. Hoechst33258 is a cell permeable DNA minor groove binder, which emits bright blue fluorescence upon excitation at 350 nm. The fluorescent confocal microscopy results show that both Hoechst33258 (14 μM, blue) and LSS-11 (5 μM, green) entered living cells and accumulated in nucleus. When cells were co-treated with both LSS-11 and Hoechst33258, the nuclei were stained by both compounds in a mutual exclusive pattern, which means Hoechst33258 binding would exclude LSS-11 binding and *vice versa* (Figure 3A). This result suggests that LSS-11 and Hoechst33258 competed with each other to bind with the chromatin.

The result was further confirmed by fluorescence competition titration. As shown in Figure 3B, LSS-11 concentration-dependently quenched the characteristic fluorescent emission of DNA-Hoechst33258 complex at 460 nm. On the other hand, LSS-11 barely had any effect on the fluorescence of EB complexed with DNA, a typical DNA intercalator, even at higher concentrations than required to quench Hoechst33258-DNA fluorescence (Figure 3C). Furthermore, as shown in Figure 3D, even 50 μM of LSS-11 showed no significant quench of the fluorescence of EB-stained pUC19 plasmid DNA in agarose gel electrophoresis. However, higher concentrations of LSS-11 did result in a visible mobility shift of electrophoresis bands, indicating binding of LSS-11 to plasmid DNA. Moreover, no cleavage of plasmid DNA by LSS-11 at tested concentrations was observed after incubated for 6 h at 37°C. Taken together, the above experimental data suggested that LSS-11 bound DNA *in vitro* and *in cell* mainly by minor groove binding, but did not cleave DNA by itself.

### LSS-11 inhibits topoisomerase II, DNA polymerase and minimal TA promoter-drove luciferase reporter expression

**Figure 4:**
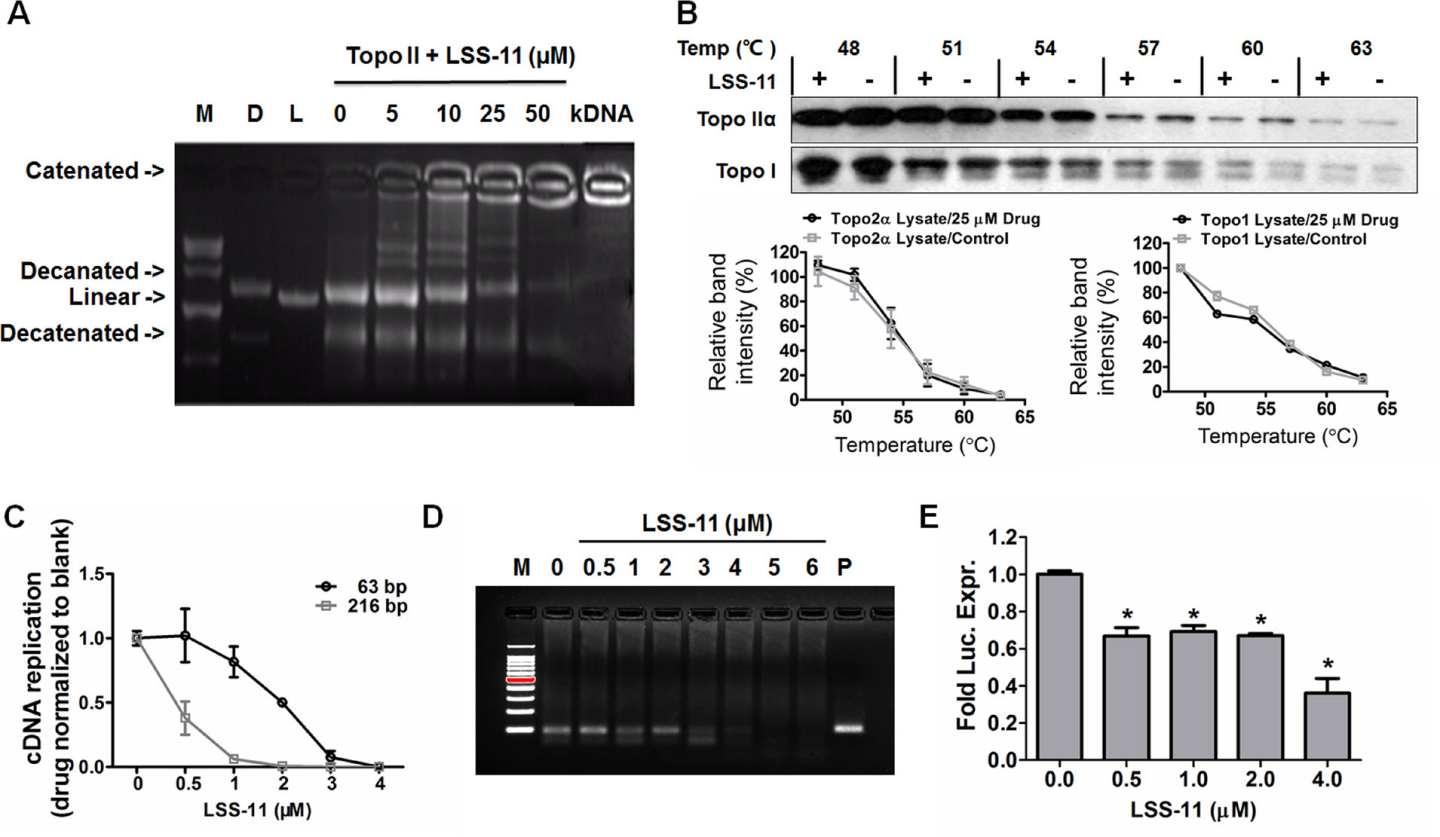
LSS-11 inhibits DNA polymerase, topoisomerase II and minimal TA promoter-drove luciferase reporter expression (**A**) The impact of indicated concentrations of LSS-11 on decatenation of kDNA by Topo II. “D” refers to decatenated DNA, “L” means linear DNA, kDNA without any treatment was included as a blank control. (**B**) The impact of LSS-11 on the thermal stabilities of Topo IIα and Topo I determined by CETSA as described in Materials and methods. Quantification of band intensity was performed by ImageJ and shown as bar graph in the lower panel. (**C**) The impact of LSS-11 on the amplification efficiency of Taq DNA polymerase in real time fluorescent quantitative PCR. 63 bp and 216 bp represent the sizes of amplicons. (**D**) The impact of LSS-11 on the efficiency of isothermal helicase-dependent amplifications. Lane M, marker; lane P, positive control. (**E**) Relative luciferase activities of SW480 cells transfected with pGL-6 TA luciferase reporter, the luciferase activity (arbitrary unit) was normalized by the luciferase activity of cells treated with vehicle only. *p < 0.05; compare with control cells.

Naphthalimides are known topoisomerase inhibitors. The effect of LSS-11 on the decatenation activity of topoisomerase II was determined using human topoisomerase II assay kit. The highly knotted circulated kDNA was retained in the sampling well in agarose electrophoresis due to extremely high molecular weight. In the presence of topoisomerase II, kDNA was decatenated into open circular and relaxed minicircle DNA. As shown in Figure 4A, LSS-11 concentration-dependently inhibited the decatenation of kDNA by topoisomerase II, with an IC_50_ of about 10 μM. On the other hand, direct binding of small molecules to a protein will change the thermal stability of the target protein. However, CETSA results indicated that LSS-11 did not alter the thermal stability of topoisomerase I and IIα in cell lysates (Figure 4B), indicating that direct interaction between LSS-11 and topoisomerases is less possible.

We used real time fluorescent quantitative PCR and isothermal HDA methods to evaluate the impact of LSS-11 on *in vitro* DNA replication. As shown in Figure 4C, LSS-11 concentration-dependently inhibited the amplification of two DNA fragments (63 bp and 216 bp) by Taq DNA polymerase. Interestingly, the inhibition on amplification of longer DNA fragment was much more potent than on that of shorter one (IC50 0.4 vs 2 μM). Meanwhile, as visualized in thermal denaturation assay, LSS-11 at lower concentrations (4 μM and lower) did not inhibit the fluorescence of SYBR green (Supplementary Figure 2). In comparison, amonafide, etoposide and SN-38 showed no observable inhibition on *in vitro* DNA replication by Taq enzyme at tested concentrations (Supplementary Figure 3A). PCR requires thermal cycling which is far from *in vivo* DNA replication, therefore isothermal HDA was employed to confirm the inhibition of DNA replication by LSS-11. As shown in Figure 4D, LSS-11 inhibited the isothermal helicase-dependent amplification of DNA, indicating the inhibition is independent of thermal cycling.

Gene transcription is another important DNA-related physiological process. The effect of LSS-11 on luciferase expression was investigated by using a pGL6-TA luciferase reporter-transfected SW480 human colorectal carcinoma cells. LSS-11 significantly inhibited the expression of luciferase driven by a minimal TA promoter, while amonafide even slightly increased the luciferase activity (Figure 4E and Supplementary Figure 3B).

### LSS-11 inhibits viability of colorectal cancer cells through apoptosis and cell cycle arrest

**Figure 5:**
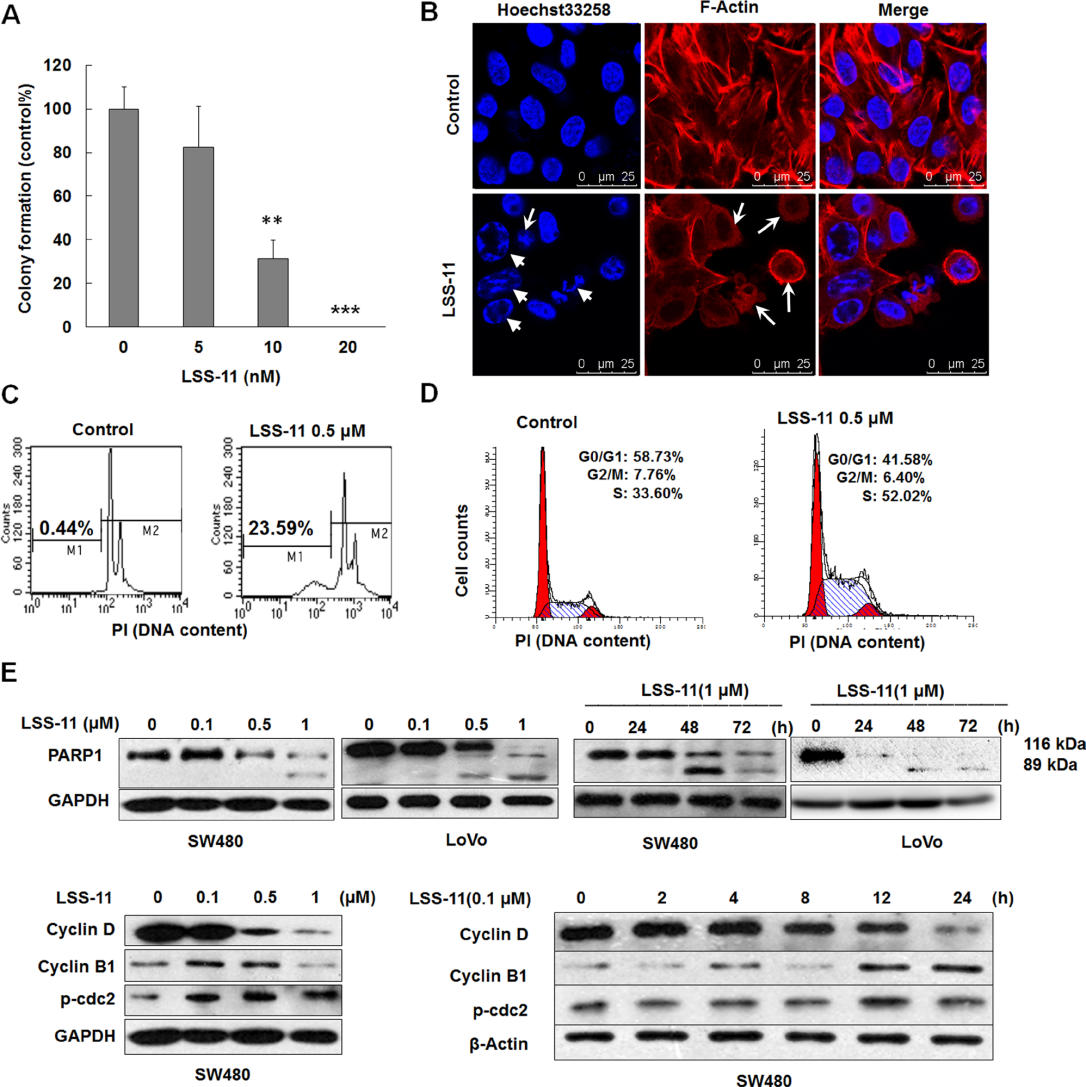
LSS-11 inhibits viability of colorectal cancer cells through apoptosis and cell cycle arrest (**A**) Colony formation ability of SW480 cells treated with indicated concentrations of LSS-11 for 14 days. **p < 0.01; ***p < 0.0001. (**B**) Confocal microscopic observation of SW480 cells treated with 2 μM of LSS-11 for 24 h, F-actin and nucleus were stained by TRITC-phalloidin and Hoechst33258, respectively. Disruption of cytoskeleton and cell shrinkage are indicated by arrows, while nuclear fragmentation and chromatin condensation are indicated by triangles. (**C**) and (**D**) Flow cytometric analyses of SW480 cells treated with 0.5 μM LSS-11 for 72 h to show sub-G1 apoptotic cells (C) and cell cycle distribution (D). Please note the × axis in (C) is exponential and × axis in (D) is linear. (**E**) and (F) Cells were treated with different concentrations of LSS-11 for indicated time, then harvested and blotted by specified antibodies, GAPDH was blotted as an internal control to assure equal loading.

The cytotoxicities of LSS-11 in HCT116, LoVo, and SW480 human colorectal cancer cell lines and HEK293 human embryonic kidney cells were determined by MTT assay. As shown in Table 1, LSS-11 time-dependently inhibited the viabilities of tested colon cancer cells with IC50 as low as tens of nanomoles after 72 h treatment. On the other hand, the IC_50_ was more than 10 folds higher in HEK293 cells than in cancer cells. The results imply that LSS-11 to some extent selectively inhibited viability of cancer cells while spared non-cancerous cells. Furthermore, LSS-11 at a dosage as low as 10 nM significantly inhibited colony formation of SW480 cells after 14 days, suggesting it possess potent anti-tumor activity (Figure 5A).

**Table 1:** Cytotoxicity of LSS-11 in selected human colon cancer cells and HEK293 cells

Time	Cell line	HCT116	LoVo	SW480	HEK293
**24 h**	IC50 (μM)	2.89 ± 0.84	0.43 ± 0.09	2.02 ± 0.45	N.D.
	Max inhibition (%)	72.30 ± 3.81	39.02 ± 2.93	61.74 ± 5.38	
**48 h**	IC50 (μM)	0.65 ± 0.2	1.40 ± 0.19	0.18 ± 0.08	12.2 ± 7.6
	Max inhibition (%)	77.45 ± 5.97	67.74 ± 2.43	69.08 ± 3.66	38.29 ± 8.02
**72 h**	IC50 (μM)	0.08 ± 0.02	0.12 ± 0.03	0.02 ± 0.01	4.81 ± 3.04
	Max inhibition (%)	93.69 ± 2.52	64.58 ± 1.23	96.22 ± 9.21	57.88 ± 10.91

Indicated cells were exposed to various concentrations of LSS-11 for 24, 48 or 72 h then the cell viabilities were determined by MTT assay. IC50 and maximum inhibition were calculated by Origin8.0 software using Hill function. Each value represents means ± SD of at least three independent experiments.

Apoptosis and cell cycle arrest are important causes for loss of viability. As shown by Hoechst33258/TRITC-phalloidin staining, LSS-11 treatment resulted in a typical apoptotic morphology, characterized by disruption of cytoskeleton, cell shrinkage, nuclei fragmentation and chromatin condensation (Figure 5B). Apoptotic DNA fragmentation was further confirmed by PI staining and flow cytometric analysis, in which LSS-11 treatment significantly increased the proportion of apoptotic cells with lower DNA content (sub-G1 peak) due to cleavage and fragmentation of genomic DNA (Figure 5C). At the same time, LSS-11 treatment significantly arrested SW480 cells in S phase cell cycle at a concentration of 0.5 μM (Figure 5D).

PARP1 is a protein involved in DNA repair and apoptosis. Upon cleavage by caspases, the smaller fragment of PARP1 binds to damaged DNA and inhibits DNA repair, thus facilitates apoptosis [29]. As shown in Figure 5E, LSS-11 induced the cleavage of PARP1 in a concentration- and time-dependent manner. The cleavage was evident at a concentration of 0.5 μM and as early as 24 h. Meanwhile, LSS-11 concentration- and time-dependently inhibited the protein levels of cyclin B and cyclin D1, while increased the phosphorylation of cdc2 at Thr14/Tyr15. These results are well correlated with the occurrence of apoptosis and cell cycle arrest. The effects of LSS-11 on the cleavage of LC3-I to LC3-II and the protein levels of p62, ATG3/5/12 and beclin 1 were also detected by western blotting, but no significant alteration was observed, indicating that autophagy was not involved in LSS-11-induced cell death (data not shown).

### LSS-11 induces DNA damage response

**Figure 6:**
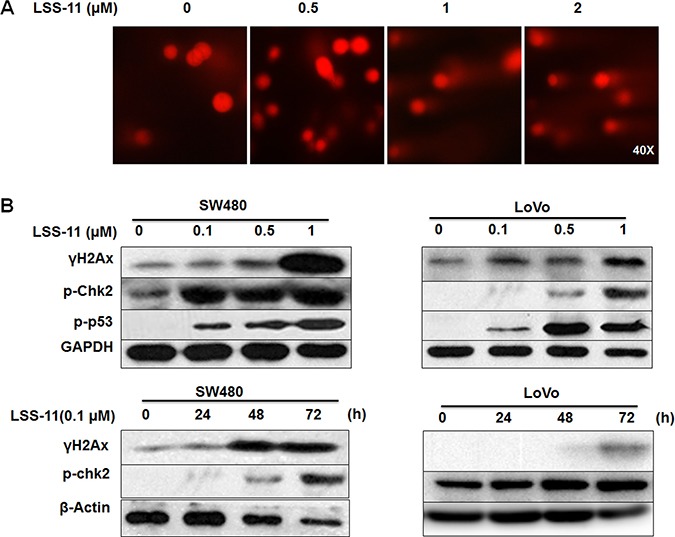
LSS-11 induces DNA damage and DNA damage response (**A**) Cells were treated with indicated concentrations of LSS-11 then DNA fragmentation was visualized by single cell electrophoresis as described in Materials and methods. (**B**) SW480 and LoVo cells were treated with indicated concentrations of LSS-11 for 72 h or 0.1 μM LSS11 for specified time, then harvested and blotted by antibodies against indicated proteins, GAPDH was blotted as an internal control to assure equal loading.

Both apoptosis and cell cycle arrest are typical responses to DNA damage. Indeed, LSS-11 treatment concentration-dependently induced significant DNA fragmentation as visualized by increased “comet” tails in single cell electrophoresis (Figure 6A). Furthermore, LSS-11 concentration-dependently and time-dependently increased the protein level of serine 139-phosphorylated histone H2AX (γ-H2AX), a key regulator and marker of cellular DNA damage response (Figure 6B). Several other important DNA damage response cascades such as increased phosphorylation of p53 and Chk2 were observed upon LSS-11 treatment. Similar results were obtained for LSS-11 in LoVo and SW480 human colorectal cancer cells (Figure 6B).

### LSS-11 inhibits tumor growth *in vivo* without significant toxicities

**Figure 7:**
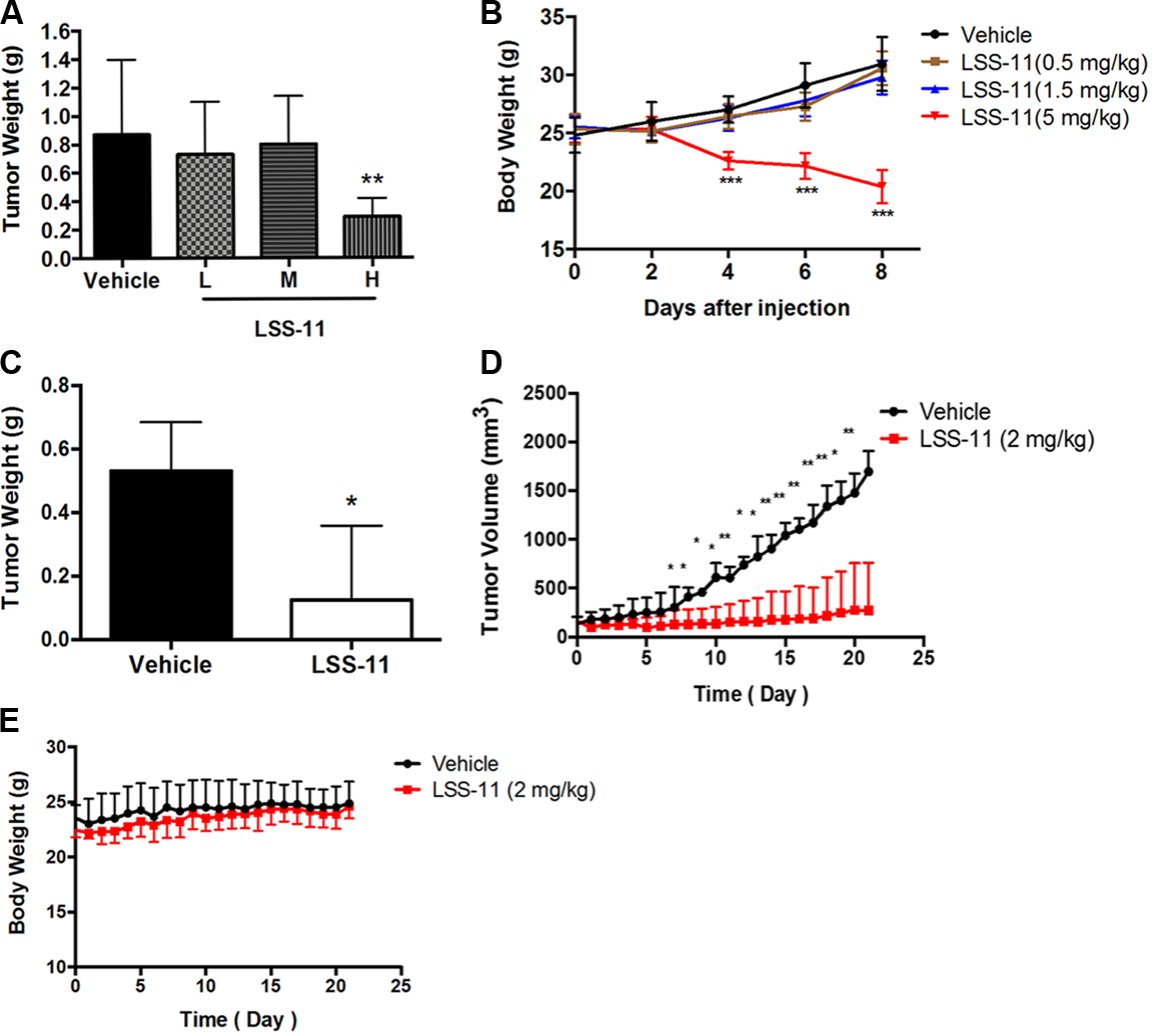
LSS-11 inhibits tumor growth *in vivo*. (**A**) The impact of LSS-11 at indicated dosages on S180 sarcoma tumor weight after 8 days’ treatment. (**B**) The impact of LSS-11 on the body weight of mice bearing S180 sarcoma. (**C**) The impact of LSS-11 (2 mg/kg) on the weight of SW480 xenografts after 3 weeks’ treatment. (**D**) The impact of LSS-11 on the growth of SW480 xenografts. *p < 0.05; **p < 0.01; ***p < 0.0001. (**E**) The impact of LSS-11 (2 mg/kg) on the body weight of Balb/C nude mice bearing SW480 xenografts.

The *in vivo* antitumor activities of LSS-11 were evaluated by using S180 sarcoma-bearing mice and SW480 xenograft nude mice models. As shown in Figure 7A and 7B, though LSS-11 at lower dosages (0.5 or 1.5 mg/kg) did not inhibit S180 sarcoma growth, it showed significant inhibition (*p* < 0.01) and reduced tumor weight at higher dosage (5 mg/kg) with a relative tumor inhibition rate of 66%, while it also caused significant body weight loss. Amonafide at 30 mg/kg dosage was employed as a positive control, however, it caused serious loss of body weight and all the mice died after 5 days, though the relative tumor inhibition rate was slightly higher than that of LSS-11 by the 4^th^ day. In a further experiment using SW480 xenografts in nude mice, the dosage of LSS-11 was adjusted to 2 mg/kg, and it showed a potent inhibition on the growth of SW480 xenografts with a relative tumor inhibition rate of 74%, while caused no significant loss of body weight (Figure 7C to 7E).

The possible hemato- (blood cell counts), cardio- (CK and αHBDH), hepato- (ALT, AST, T-Bil and LDH), neuro- (plantar test), and renal (Cre and Urea) toxicities of LSS-11 in mice were preliminarily evaluated by organ weight, whole blood cell count, serum biochemical analysis and plantar test. As shown in Supplementary Table 1 and Supplementary Figure 4, only lymphocyte counts exhibited a mild but significant (*p* = 0.05) elevation (2.36 ± 0.76 vs. 3.7 ± 1.13) after treated with 2 mg/kg of LSS-11 for 21 days. No other significant adverse effect was observed.

## DISCUSSION

Naphthalimides are typical DNA-targeting compounds that have been extensively explored as anti-cancer agents [20], and several of them have successfully achieved clinical trials; however, all failed due to unfavorable toxicities and/or limited therapeutic efficacy [21]. Novel naphthalimides with improved efficacy and toxicology have received enormous research interests, and by chance we synthesized a class of triazolenaphthalimide derivatives which exhibited extraordinary cytotoxicities in a panel of cancer cell lines, especially colon cancer cells [25]. LSS-11, characterized by a fused triazole ring to the naphthalene core and a tertiary amine moiety attached to the triazole ring by three methylene groups (Figure 1), is one of the most potent compounds [26]. As shown in Table 1 and to our best knowledge, it is one of the most potent anti-proliferative naphthalimides against solid tumors that have been reported. The cytotoxicity of LSS-11 was most pronounced after 72 h, but even at 24 or 48 h it was still better than amonafide [9]. The time dependency was further manifested by the result of colony formation assay, in which 14 days of LSS-11 treatment significantly inhibited formation of cancer cell colonies at a concentration as low as 10 nM (Figure 5A). More importantly, LSS-11 at a dosage of only 2 mg/kg potently inhibited the growth of S180 sarcoma and SW480 xenografts *in vivo* (Figure 7), while no significant hemato-, cardio-, hepato-, peripheral neuro- and renal toxicities except for a mild increase of lymphocytes were observed after LSS-11 treatment for 21 days (Supplementary Table 1 and Supplementary Figure 4). These results suggest LSS-11 deserves further investigation and development.

Cell cycle arrest and apoptosis are the most common cellular mechanisms by which naphthalimides and other DNA-targeting agents inhibit cancer cell growth [30]. As expected, LSS-11 induced significant S phase cell cycle arrest and apoptosis at a concentration of 0. 5 μM, which are also evidenced by the time- and concentration-dependent inhibition of cyclins expression and cleavage of PARP-1 (Figure 5). DNA damage will cause cell cycle arrest and apoptosis, and several naphthalimides have been reported to induce DNA strand breaks both *in vitro* and *in vivo* [31, 32]. LSS-11 did not induce cleavage of super coiled plasmid DNA *in vitro*; however, it significantly induced DNA fragmentation in cells, as demonstrated by comet assay and flow cytometric analysis (Figure 3D and Figure 6A and Figure 5C). Furthermore, LSS-11 treatment induced significant elevation of phosphorylated H2AX (γ-H2AX), p53 and Chk2 (Figure 6B). Phosphorylation of H2AX by ATM/ATR kinases is the first step to recruit DNA repair enzymes, and p53 and CHK2 are phosphorylated by the same upstream kinases in response to DNA damage or replication stress and initiate S phase checkpoints to ensure genome integrity [33]. Actually, inhibition of topoisomerases or DNA replication is the most common cause of DNA damage response, and cancer cells that have loose cell cycle control and more genome instability are more susceptible to DNA damage responses [34]. Therefore, our results indicate that LSS-11 elicited replication stress and/or DNA damage responses, which may be responsible for the S-phase arrest and apoptosis induced by LSS-11.

DNA is the most exploited target of chemotherapeutic agents, and binding to DNA has been suggested as a prerequisite for the cytotoxicity of naphthalimides [22]. LSS-11 bound to DNA with a moderately high affinity (about 2.5 × 10^5^ M^-1^, Figure 2); in comparison, the association constants of amonafide and bis-naphthalimides (well known for their high DNA binding affinity) are about 1.5 × 10^5^ M^-1^ and 10^6^ M^-1^, respectively [20]. Though amonafide and most naphthalimides are generally regarded as DNA intercalators, LSS-11 bound to DNA mainly by minor groove binding, since it competed with Hoechst33258 rather than EB to bind with DNA (Figure 3). On the other hand, the binding of LSS-11 to RNA suggests the interaction depends on neither the B-form DNA double helix nor intercalation, since RNA only partially forms double helix in A-form geometry. Actually, several naphthalimides have been confirmed to bind with DNA by minor groove binding [19, 28]. In the case of LSS-11, the intercalation of the aromatic rings could be hindered by the amino side chain attached to the triazole ring (Figure 1). Possibly binding of LSS-11 to DNA minor groove provides more opportunities to approach DNA-protein interactions than intercalation does, especially with the triazole moiety and amino side chain [35, 36]. Indeed, it has been reported that minor groove binding interferes the binding of RNA polymerase II and transcription factors, thus represses transcription process [37]. Furthermore, LSS-11 predominantly accumulated in cell nucleus (Figure 3A). These results suggest that DNA was the primary target of LSS-11 in cells.

Binding of small molecules to DNA in most cases results in perturbation of DNA structure and interferes with DNA-protein interactions, thus modulates DNA-associated physiological processes including but not limited to replication, transcription, repair, recombination, etc., and each process involves specific sets of enzymes/proteins [4]. Topoisomerases, a class of the most important chemotherapeutic targets, can resolve topological entanglement of DNA strands and are involved in almost all types of DNA transactions [38]. Topo II is the most well studied target of naphthalimides [39], and our experimental results also demonstrate that LSS-11 inhibited human Topo II with an IC50 of about 10 μM (Figure 4A). The inhibitory activity is comparable to that of bis-naphthalimide DMP-840, and significantly more potent than that of amonafide (~357 μM) [40]. Unlike other Topo II poisons, LSS-11 did not directly interact with Topo II enzymes as demonstrated by CETSA result, suggesting that it inhibited Topo II mainly by targeting DNA or protein-DNA interactions (Figure 4B). It is worthy note that it requires much higher concentrations of LSS-11 for Topo II inhibition than for other activities such as cytotoxicity, cell cycle arrest and apoptosis, and to elicit other molecular events, and suggests additional mechanisms are involved in their cytotoxicities.

Triazole modification of DNA has been found to facilitate RNA synthesis by *in vitro* translation [41]. In contrast, LSS-11 but not amonafide significantly inhibited the *in vivo* expression of a luciferase reporter driven by a minimal TA promoter, suggesting LSS-11 might inhibit translation of DNA in cells (Figure 4E and Supplementary Figure 3B). DNA replication is another important DNA-associated process that has been proposed as a potential target of anti-cancer therapies [42]. A few studies have investigated direct inhibition of DNA polymerases. For example, cisplatin and actinomycin D have been reported to inhibit DNA synthesis *in vitro* [43, 44]. Here we utilized isothermal HDA and Taq DNA polymerase-mediated qPCR to quantify the inhibition of DNA synthesis by LSS-11. LSS-11 potently inhibited DNA amplification at much lower concentrations than required to inhibit Topo II, while amonafide did not show any inhibition (Figure 4E and Supplementary Figure 3B). Interestingly, the inhibitory effect of LSS-11 was more potent for longer DNA fragment (216 bp, IC50 ~ 0. 4 μM) than shorter one (63 bp, IC50 ~ 2 μM), possibly because the longer DNA could bind more LSS-11 molecules. It is reasonable to expect an even more potent inhibition if we extrapolate the DNA fragments to genomic DNA in cell nucleus. The above data demonstrated that LSS-11 inhibited almost all DNA-associated transactions, and the integrated effects of LSS-11 on DNA and associated processes may be responsible for its potent cytotoxicity, since the concentrations required to inhibit Topo II, DNA replication and transcription were still higher than that required to inhibit cancer cell proliferation. As a matter of fact, many topoisomerase inhibitors require much higher concentrations to inhibit topoisomerases even in *in vitro* experiments than that required to inhibit cancer cell proliferation [35]. Such discrepancy has also been observed for other FDA-approved topoisomerase inhibitors such as etoposide (https://pubchem.ncbi.nlm.nih.gov/assay/bioactivity.html?cid=36462) and SN-38 (https://pubchem.ncbi.nlm.nih.gov/assay/bioactivity.html?cid=104842, the active metabolite of irinotecan). In addition, other targets such as DNA in mitochondria could also be involved in the cytotoxicities of naphthalimides and topoisomerase inhibitors, since in most cases cytotoxicity was determined by MTT which measures mitochondrial succinate dehydrogenase activity. Indeed, recently naphthalimide derivatives have been reported to inhibit metastasis by targeting mitochondria [45]. Our results provide new possible interpretations of such paradoxical observations.

In conclusion, here we reported a novel triazolonaphthalimide, LSS-11, with improved anti-cancer efficacy and tolerability, and explored the underlying cellular and biochemical mechanisms. The present study provides novel mechanistic insight into the anti-cancer activities of naphthalimides, and suggests that LSS-11 deserves further research and development as a novel anti-cancer agent.

## MATERIALS AND METHODS

### Chemicals and reagents

Antibodies against cyclin B1, cyclin D, p-cdc2, PARP, γH2Ax and p-chk2 were obtained from Cell Signaling Technology (Beverly, MA). Antibodies against β-Actin, β-Tubulin, GAPDH and horseradish peroxidase (HRP)-conjugated secondary antibodies were purchased from Bioeasy Tech (Beijing, China). Antibodies against Topo IIα and Topo I were purchased from ABclonal Biotech (College Park, MD). IsoAmp II universal tHDA kit was obtained from New England Biolabs Inc. (Ipswich, MA, USA). Luciferase assay substrate and pGL6-TA Luciferase reporter which encodes firefly luciferase driven by a minimal TA promoter were purchased from Beyotime Institute of biotechnology, Jiangsu, China. SYBR Green qPCR Mix was obtained from AidLab Biotech, Beijing, China. Calf thymus DNA (CT DNA), 3-(4,5- dimethylthiazoyl-2-yl) 2,5-diphenyltetrazolium bromide (MTT), propidium iodide (PI), ethidium bromide (EB) and Hoechst33258 were obtained from Sigma-Aldrich (St. Louis, MO). Culture media, Lipofectamine 2000 and TRITC-phalloidin were purchased from Life Technologies (Carlsbad, CA). Etoposide and SN-38 were purchased from Selleck Chemicals (Huston, TX). All the other chemicals were of the highest grade available.

2-amino-5-(2-(dimethylamino)ethyl)-10-(3-(dimethylamino) propyl) benzo [*de*] [1,2,3] triazole [4,5-g] isoquinoline-4,6(5*H*,10*H*)-dione (LSS-11) with a purity > 95% was synthesized and characterized as previously described [25, 26]. LSS-11 was dissolved in DMSO to make a stock concentration of 100 mM and diluted to indicated concentrations before use.

### DNA thermal denaturation assay

A modified SYBR Green-based real time fluorescent quantitative polymerase chain reaction (qPCR) method was utilized to detect the impact of LSS-11 on the melting temperature (Tm) of DNA. A 63 bp DNA fragment was amplified by PCR using SYBR Green qPCR Mix (AidLab Biotech, Beijing, China) and cDNA template with specific primers (Table 2), then the resulted DNA product was equally aliquoted and incubated with indicated concentrations of LSS-11 for 30 min at room temperature. After that the melting curves and Tm values were determined by a Bio-Rad CFX Connect Real-Time PCR Detection system (Bio-Rad Laboratories, Berkeley, CA) at 1°C/cycle increment.

**Table 2:** The information of primers used in the present study

Gene symbol	Sequences (5′–3′)	Amplicon size (bp)	GC%	Calculated Tm (°C)
NFE2L2	F: TCAGCATGCTACGTGATGAAG	63	43%	74.56
	R: TTTGCTGCAGGGAGTATTCA			
ABCC2	F: TCCCTGTCCCTAGGGCTTTT	216	39%	80.01
	R: CTGCGTCTGGAACGAAGACT			
GAPDH	F: ACTCTGGTAAAGTGGATATTGTTGCCA	95	41%	75.68
	R: TTTGCCATGGGTGGAATCATATTGGAA			

### Ultraviolet-visible (UV-vis) and fluorescent spectra titration

50 μM of LSS-11 solution in Tris-HCl buffer (30 mM, pH 7.5) was incubated with increasing concentrations ranging from 0 to 200 μM of CT DNA at room temperature for 2 h, then the UV-vis absorption spectra were recorded using a Varian Cary-300 Spectrophotometer (Agilent Technologies, Santa Clara, CA). The molar concentrations of CT DNA were calculated as single nucleotide. Because the absorption of DNA at 260 nm was overlapping with the absorption of LSS-11, the absorbance of LSS-11 at 225 nm was used to calculate the binding affinity of LSS-11 to DNA according to published method [46]. The excitation wavelength was fixed at 386 nm and the fluorescent spectra of LSS-11 between 400–770 nm were recorded on a Varian Cary Eclipse fluorescent spectrophotometer (Agilent Technologies, Santa Clara, CA).

Competitive fluorescent spectra titration was performed as previously reported [47]. 50 μM of CT DNA solution in Tris-HCl buffer (30 mM, pH 7.5) was incubated with 14 μM of Hoechst33258 or 5 μM of EB and increasing concentrations of LSS-11 (ranging from 0 to 10 μM) at room temperature for 2 h, then the fluorescent spectra of Hoechst33258 (excitation at 350 nm, emission from 380 to 600 nm) or EB (excitation at 530 nm, emission from 530 to 750 nm) were recorded on a Varian Cary Eclipse fluorescent spectrophotometer.

### Evaluation of DNA replication efficiency by quantitative PCR

Firstly, Taq DNA polymerase-mediated SYBR green-based real time fluorescent qPCR method was employed to quantitatively determine the impact of LSS-11 on DNA replication. CDNA was prepared from SW480 cells by using a RevertAid First Strand cDNA Synthesis Kit (Thermo Fisher Scientific, Waltham, MA) following manufacturer's instructions and used as template of all following PCR assays. Two DNA fragments were amplified using two pairs of primers for NFE2L2 and ABCC2, which generate 63 bp and 216 bp amplicons, respectively (Table 2), and the fluorescence was monitored by a Bio-Rad CFX Connect Real-Time PCR Detection system (Bio-Rad Laboratories, Berkeley, CA) following standard procedure. The relative amplification efficiency was calculated using comparative quantification cycle (Cq) (ΔCq) method using vehicle control without LSS-11 as reference.

### Thermophilic helicase-dependent amplification (tHDA)

Isothermal helicase-dependent amplification (HDA) assay was further performed to evaluate the impact of LSS-11 on DNA replication efficiency without thermal cycling. A 95 bp GAPDH fragment was amplified using an IsoAmp^®^ II Universal tHDA Kit (New England Biolabs Inc., Ipswich, MA, USA) and specific primers (Table 2) following manufacture's two-step reverse transcription-HDA protocol. Briefly, 0.2 μg cDNA template was incubated with annealing buffer and primers for 2 min at 95°C, then the above mix was cooled on ice and mixed with the enzymes mix containing ThermoScript reverse transcriptase, IsoAmp^®^ dNTP and enzymes in annealing buffer. Following incubation for 120 min at 65°C on a VeritiTM 96 well Thermal cycler (Applied Biosystems, Foster City, CA, USA), the products were separated by 2% gel staining with SYBR green and the bands were quantified by ImageLab software (Bio-Rad Laboratories, Berkeley, CA).

### Topo II activity assay

Topo II activity was measured by the ATP-dependent decatenation of highly knotted circulated DNA (kDNA) following the protocol provided by the manufacturer (TopoGEN, Florida, USA). Briefly, 0.25 μg kDNA and 1 unit of recombination Topo IIa enzyme were incubated in reaction buffer at 37°C for 30 min in the presence of LSS-11 ranging from 0~50 μM, then stopping buffer and 20 μg/mL proteinase K were added and incubated for further 15 min. Previously decatenated kDNA was used as a positive control, and kDNA without Topo II was included as a blank control. Samples were then separated by agarose gel electrophoresis and visualized by EB staining. The optical density was quantified by a Bio-Rad Quantity One software, and THE half maximal inhibitory concentration (IC50) was calculated by Scatchard plot analysis.

### Luciferase reporter expression assay

SW480 cells were transfected with 0.3 μg of pGL6-TA luciferase reporter plasmid per well using Lipofectamine 2000 transfection reagent following the manufacturer's instructions (Life Technologies, Carlsbad, CA), then cultured for additional 24 h. The cells were treated with different concentrations of indicated compounds for 6 h, washed by cold PBS and collected in reporter lysis buffer on ice for 10 min. Lysates were centrifuged at 8000 rpm and 50 μl of supernatant was used for luciferase activity measurement using a Synergy H1 multi-mode reader (Bio-Tek Instruments, Winooski, Vermont). The obtained reads were normalized by protein concentrations measured using BCA method, and the normalized luciferase activities were expressed as folds of control.

### Cellular thermal shift assay (CETSA)

The potential interaction between LSS-11 and topoisomerases was assessed by CETSA as previously described [48]. Briefly, cells were harvested in RIPA buffer with complete protease inhibitors and freeze-thawed three times using liquid nitrogen. The lysate was centrifuged at 12000 rpm for 20 minutes at 4°C, and the supernatant was divided into two equal parts to incubate with LSS-11 or DMSO as vehicle for 30 min at room temperature. The resulted aliquots were then divided into smaller parts and heated at indicated temperatures using a gradient thermal cycler (T-100, Bio-Rad) for 3 minutes. The heated lysates were centrifuged at 12000 rpm for 20 minutes at 4°C to separate the soluble fractions and boiled at 95°C with 5× SDS loading buffer for 5 min and analyzed by western blotting.

### Cell culture and treatments

SW480, LoVo, and HCT116 human colon cancer cells were obtained from and validated by Chinese Academy of Medical Sciences (Beijing, China). HEK293 human embryonic kidney cells were obtained from American Type Culture Collection (Manassas, VA). The cells were cultured in IMDM (for colon cancer cells) or MEM (for HEK293 cells) supplemented with 10% FBS in a humidified 5% CO_2_ atmosphere at 37°C. All experiments were performed using cells in exponential growth within 20 passages, the origination and homogeneity have been authenticated by short tandem repeat (STR) analysis. The cells were cultured in culture plates or dishes to 70%–80% confluence, then the media were replaced by complete media containing various concentrations of LSS-11 for indicated time, and 0.1% DMSO was employed as vehicle control.

### Cytotoxicity assay

Cells were cultured in 96-well plates and treated with various concentrations of LSS-11 for 24, 48 or 72 h, then MTT was added into medium to final concentration of 0.5 mg/mL and incubated for another 2 h, then the media were removed and the resulted formazan precipitates were dissolved in DMSO, and the optic density at 492 nm was read immediately using a MultiSkan MK3 microplate reader (Thermo Fisher Scientific, Waltham, MA). The IC50 and maximum inhibition rate were calculated by Origin 8.0 software using Hill function.

### Colony formation assay

SW480 cells were seeded in 6-well plates at a density of 200 cells/well, then treated with indicated concentrations of LSS-11 in complete medium for 14 days to allow the formation of colonies, during the period the medium was changed every three days. After the treatment, the cells were fixed with glutaraldehyde (6% w/v) for 60 min, followed by crystal violet (0.5% w/v) staining. The stained colonies were counted on microscope, and the colony formation rate was calculated as percentage of the control group. The results are presented as the mean ± SD of three independent experiments.

### Flow cytometry analysis

The cells were treated with indicated concentrations of LSS-11 for 72 h, then both the floating and attached cells were harvested and fixed in ice-cold 70% alcohol. Fixed cells were washed twice by cold PBS, then incubated in staining solution (5 μg/ml propidium iodide, 100 μg/ml RNase, 0.2% Triton X-100) at 37°C for 30 min, the cellular DNA content was analyzed on a BD FACScalibur flow cytometer (BD bioscience, San Jose, CA), and the cell cycle distribution and percentage of apoptotic cells were calculated using BD CellQuest Pro software.

### Single cell gel electrophoresis (Comet assay)

Single cell gel electrophoresis was performed as previously reported [49]. The cells were treated with indicated concentrations of LSS-11 for 48 h, then harvested by trypsinization, mixed with 1% low-melting point agarose, layered onto microscope slides pre-coated with normal-melting point agarose. The cells in the gel were permeabilized in 1%Triton-X buffer (100 mM EDTA, 2.5 M NaCl, 10 mM Tris, pH 10.0) at 50°C for 1 h, then lyzed in alkaline solution (0.3 M sodium hydroxide, 10 mM EDTA, pH >13.0). The gels were electrophoresed and neutralized in Tris buffer (10 mM Tris-HCl, 10 mM EDTA, pH = 7.5), stained by 50 μg/mL EB, and visualized on a Leica SP5 II laser scanning confocal microscope system (Leica Microsystems, Wetzlar, Germany).

### Fluorescent microscopy

The cells were cultured on glass coverslips and allowed to grow for 24 h. To visualize the competitive binding of Hoechst33258 and LSS-11 to DNA in cell nucleus, the living cells were directly incubated with 14 μM of Hoechst33258 or 5 μM of LSS-11 or both for 1 h at 37°C. To examine the apoptotic cellular morphology induced by LSS-11 treatment, the cells were treated with LSS-11 for 24 h as described above. The treated cells were fixed by 4% formaldehyde, permeabilized by 0.2% Triton X-100, then stained in PBS containing 5 μg/ml Hoechst33258 and TRITC-phalloidin for 30 min at room temperature. The fluorescently stained cells were then photographed on a Leica SP5 II laser scanning confocal microscope system (Leica Microsystems, Wetzlar, Germany).

### Western blotting

Cells were treated with indicated concentrations of LSS-11 for indicated time, then harvested in RIPA buffer. The cell lysates were collected and centrifuged at 4°C for 12000g to remove unsolved debris, and the protein concentrations were determined by BCA assay. Aliquots containing same amounts of proteins were resolved by 8%~15% SDS-PAGE, then proteins were electro-transferred to PVDF membrane. And blocked by 5% BSA in phosphate-buffered saline-0.1% Tween 20, the membrane was probed with appropriate primary antibodies at 4°C overnight, then washed and incubated with corresponding HRP-conjugated secondary antibodies at room temperature for 1 h. The blots were then washed and visualized by ECL™ Prime Western Blotting detection reagent (Amersham-Pharmacia, Piscataway, NJ) and Kodak X-ray films.

### *In vivo* anti-tumor activity evaluation

All animals were obtained from and housed in the Department of Laboratory Animal Science of Peking University Health Science Center on a 12 hrs light/dark cycle under controlled temperature and humidity, with access to standard diet and water *ad libitum*. All experimental protocols have been approved by the Peking University Institutional Animal Care and Use Committee (IACUC).

Male ICR mice weight 17–19 g were hypodermically inoculated with 3 × 10^6^ cells/mouse of S180 murine sarcoma cells and allowed to grow for 1 week. The mice were divided into 5 groups according to body weight: model (saline only), amonafide (30 mg/kg), LSS-11 (0.5, 1.5, 5 mg/kg), then treated with drugs or saline by intraperitoneal injection for 7 consecutive days. The body weights were recorded every two days. All animals in amonafide group died at 5th day. The rest of the mice were then sacrificed at the end of experiments and sarcoma tumors were excised, photographed and weighted, and the relative tumor inhibition rate was calculated.

In another experiment, 3 × 10^6^ SW480 cells were inoculated into right flank of male Balb/C (nu/nu) mice aged 6 weeks. Tumor size was calculated as length × width^2^/2. The mice were randomly divided into two groups when the tumor size reached 100 mm3, then treated with saline or LSS-11 (2 mg/kg) by intraperitoneal injection for 21 consecutive days. The tumor size and body weight were measured every day, and at the end of experiment the mice were sacrificed and the xenografts were excised and weighted, and the relative tumor inhibition rate was calculated.

### Statistics

All data are shown as mean ± standard deviation (S.D.) using two-tailed Student's *t* test and one-way analysis of variance with Bonferroni multiple comparison post-test. *p* < 0.05 was considered as a significant difference.

## SUPPLEMENTARY MATERIALS FIGURES AND TABLES


