# Supplementary material for: A novel triazolonaphthalimide induces apoptosis and inhibits tumor growth by targeting DNA and DNA-associated processes
Source: Oncotarget. 2017 Apr 8;8(23):37394–408. doi: 10.18632/oncotarget.16962 (PMC5514917; doi:10.18632/oncotarget.16962)
Supplement: Supplementary file 1 [file oncotarget-08-37394-s001.pdf]

A novel triazolonaphthalimide induces apoptosis and inhibits tumor growth by targeting DNA and DNA-associated processes

Supplementary Materials

SUPPLEMENTARY MATERIALS AND METHODS

In vivo toxicity evaluation

Male ICR mice weight 17–19 g were intraperitoneally given saline (control), 2 mg/kg LSS-11 for 21 days. The body weight was recorded every two days. At the end, mice were sacrificed, and lung, heart, liver, kidney and spleen were collected and weighted. Blood was collected for complete blood cell count by hematology analyzer (Nihon Kohden, Tokyo, Japan). Serum levels of aspartate, alanine transaminase (AST and ALT), total bilirubin (T-Bil), lactate dehydrogenase (LDH), creatine kinase (CK), alpha-hydroxybutyrate dehydrogenase (αHBDH), creatine, and urea were measured by an AU5400 automatic biochemical analyzer (Hitachi Corp., Japan) and Roche Diagnostics kit (Roche, USA).

Plantar test was performed after 7 days' LSS-11 treatment to determine peripheral neurotoxicity as previously reported [1]. Mice were kept in a chamber for 20 minutes to acclimate. Then, a portable heat source with 56 W infrared radiance was placed beneath the plantar surface of the paw and the latency of paw withdrawal was recorded for each mouse by a Plantar Test Instrument (Hargreave's Method) (Ugo Basile, Comerio, Italy).

REFERENCE

1. Bouchard VJ, Rouleau M, Poirier GG. PARP-1, a determinant of cell survival in response to DNA damage, Experimental Hematology. 2003; 31:446–454.

Supplementary Table 1: LSS-11 has no significant toxicities in mice

	Ctrl	LSS-11	Unit	*P* value
Body weight	32.92 ± 1.25	32.08 ± 2.41	g	0.48
Heart index	0.50 ± 0.037	0.49 ± 0.037	%	0.81
Lung index	0.65 ± 0.034	0.67 ± 0.048	%	0.45
Liver index	4.70 ± 0.30	4.64 ± 0.26	%	0.70
Kidney index	0.79 ± 0.079	0.85 ± 0.075	%	0.19
Spleen index	0.46 ± 0.03	0.43 ± 0.06	%	0.33
**Complete blood count**				
WBC	4.62 ± 2.13	5.10 ± 1.66	× 1000/mm^3	0.67
RBC	7.80 ± 0.41	7.781 ± 0.82	× 10^4/mm^3	0.96
HGB	142.8 ± 11.62	139.43 ± 18.05	g/dL	0.72
HCT	38.58 ± 3.38	37.21 ± 4.64	%	0.59
MCV	49.44 ± 3.27	47.78 ± 2.30	fL	0.33
MCH	18.32 ± 0.71	17.87 ± 0.69	pg	0.30
PLT	796.6 ± 155.94	693.43 ± 138.37	× 1000/mm^3	0.25
LYM	2.36 ± 0.76	3.7 ± 1.13	10^3/μL	0.05
MID	1.44 ± 1.93	1.1 ± 0.59	10^3/μL	0.69
GRN	0.82 ± 0.40	0.65 ± 0.28	10^3/μL	0.43
**Blood chemistry**				
ALT	43.6 ± 12.26	40.78 ± 10.84	U/L	0.66
AST	97 ± 16	109.56 ± 19.47	U/L	0.24
T-Bil	2.26 ± 0.38	2.033 ± 0.40	μmol/L	0.32
LDH	516.8 ± 104.08	552.44 ± 113.90	U/L	0.57
CK	924 ± 326.86	1191.67 ± 379.34	U/L	0.21
HBDH	188.4 ± 42.48	204.89 ± 44.68	U/L	0.51
Cre	21.4 ± 6.19	19.44 ± 3.04	μmol/L	0.44
Urea	10.72 ± 1.56	10.278 ± 0.86	mg/dL	0.50

Note: ALT, alanine transarninase; AST, aspartate aminotransferase; T-Bil, total bilirubin; LDH, lactate dehydrogenase; CK,creatine kinase; HBDH,α-hydroxybutyrate dehydrogenase; Cre,creatinine; Urea,urea nitrogen; WBC, white blood cells; RBC, red blood cells; HGB, hemoglobin; HCT, hematocrit; MCV, mean volume of erythrocytes; MCH, mean content of hemoglobin; PLT, platelets; LYM, lymphocyte; MID, mid-cells; GRN, granulocytes.
Organ index, complete blood count and blood biochemistry were analyzed in mice given by 2 mg/kg LSS-11 for 21 days.

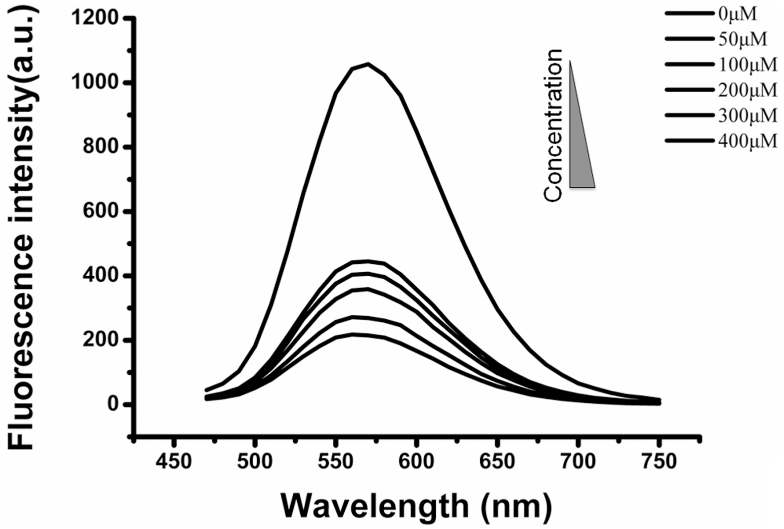

Supplementary Figure 1: LSS-11 interacts with RNA. Fluorescent emission spectra of LSS-11 (50 μM) with increasing concentrations of RNA (0, 50, 100, 200, 300, 400 μM).

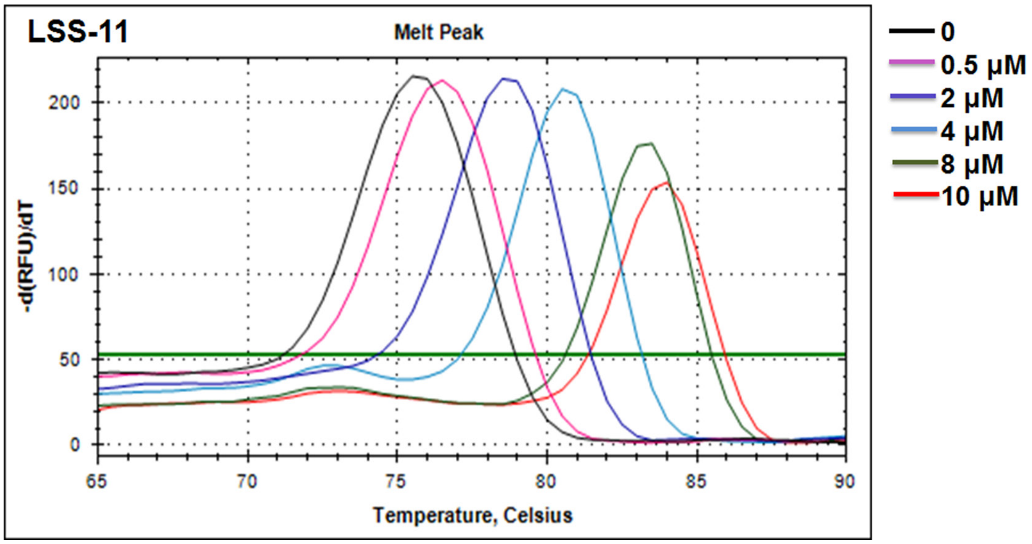

Supplementary Figure 2: Amplification melting curve of LSS-11 as detected by real time fluorescent quantitative PCR at indicated concentrations.

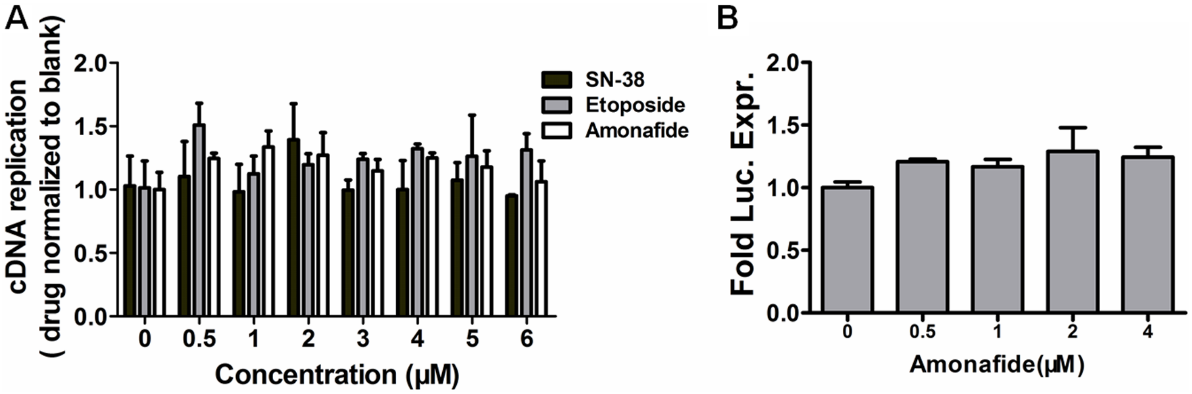

Supplementary Figure 3: The effect of known topoisomerase inhibitors on DNA replication and transcription. (**A**) The impact of SN-38, etoposide and amonafide on the amplification efficiency of real time fluorescent quantitative PCR reactions using Taq DNA polymerase. (**B**) Relative luciferase activities of SW480 cells transfected with pGL-6 TA luciferase reporter normalized by the luciferase activities of cells in the presence or absence of amonafide.

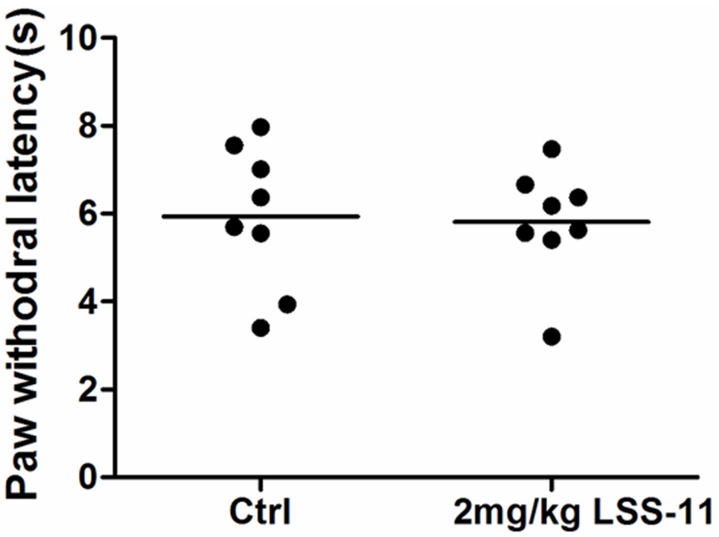

Supplementary Figure 4: LSS-11 exhibited no peripheral neurotoxicity in mice as measured by plantar thermal test.
